# 

**Published:** 1911-10-07

**Authors:** 


SPECIAL THE NUMBER.

### HOSPITAL

The Workers' Newspaper of Administrative

Medicine and Institutional Life.

Telegraphic Address: OSPEDALE, LONDON. Telephone Number: 2734 GERRARD.

No. 1317, Vol. LI. | Saturday, October 7th, 1911. | PRICE ONE PENNY

